# Exercise Intensity During Cross-Country Skiing Described by Oxygen Demands in Flat and Uphill Terrain

**DOI:** 10.3389/fphys.2018.00846

**Published:** 2018-07-09

**Authors:** Øyvind Karlsson, Matthias Gilgien, Øyvind N. Gløersen, Bjarne Rud, Thomas Losnegard

**Affiliations:** ^1^Department of Physical Performance, Norwegian School of Sport Sciences, Oslo, Norway; ^2^Norwegian Ski Federation, Alpine Skiing, Oslo, Norway; ^3^Condensed Matter Physics, Department of Physics, University of Oslo, Oslo, Norway

**Keywords:** cross-country skiing, exercise intensity, external power, global navigation satellite system, metabolic rate, pacing

## Abstract

**Purpose:** In this study wearable global navigation satellite system units were used on athletes to investigate pacing patterns by describing exercise intensities in flat and uphill terrain during a simulated cross-country ski race.

**Methods:** Eight well-trained male skiers (age: 23.0 ± 4.8 years, height: 183.8 ± 6.8 cm, weight: 77.1 ± 6.1 kg, VO_2peak_: 73 ± 5 mL⋅kg^-1^⋅min^-1^) completed a 13.5-km individual time trial outdoors and a standardized indoor treadmill protocol on roller skis. Positional data were recorded during the time trial using a differential global navigation satellite system to calculate external workloads in flat and uphill terrain. From treadmill tests, the individual relationships between oxygen consumption and external workload in flat (1°) and uphill (8°) terrain were determined, in addition to VO_2peak_ and the maximal accumulated O_2_-deficit. To estimate the exercise intensity in the time trial, the O_2_-demand in two different flat and five different uphill sections was calculated by extrapolation of individual O_2_-consumption/workload ratios.

**Results:** There was a significant interaction between section and average O_2_-demands, with higher O_2_-demands in the uphill sections (110–160% of VO_2peak_) than in the flat sections (≤100% of VO_2peak_) (*p* < 0.01). The maximal accumulated O_2_-deficit associated with uphill treadmill roller skiing was significantly higher compared to flat (6.2 ± 0.5 vs. 4.6 ± 0.5 L, *p* < 0.01), while no significant difference was found in VO_2peak_.

**Conclusion:** Cross-country (XC) skiers repeatedly applied exercise intensities exceeding their maximal aerobic power. ΣO_2_-deficits were higher during uphill skiing compared to flat which has implications for the duration and magnitude of supramaximal work rates that can be applied in different types of terrain.

## Introduction

Cross-country (XC) skiing is an endurance sport in which the goal is to cover a known distance in the shortest time possible. Unlike most other endurance sports such as track running, rowing, or swimming, a substantial variation in speed exists, since competition courses in XC skiing must consist of approximately one-third ascending, one-third flat and one-third descending terrain (FIS, 2017). The large fluctuations in speeds, imposed by the topography of the course, challenge skiers’ ability to control the exercise intensity, also described as the athletes pacing (Abbiss and Laursen, 2008).

It is widely accepted that pacing patterns have a significant influence on performance in a variety of sports, including XC skiing (Abbiss and Laursen, 2008; Losnegard et al., 2017). Theoretically, an even pacing pattern is regarded as optimal for performance in endurance sports events with durations > 2 min, where athletes race against the clock over a known distance (Abbiss and Laursen, 2008). In contrast, studies of running (Tucker et al., 2006; Hanley, 2015), cycling (Thomas et al., 2012), mountain bike (Martin et al., 2012), and rowing (Garland, 2005) have shown that athletes, in fact, apply positive, J-shaped or variable pacing patterns. Furthermore, studies on pacing patterns in XC ski racing have consistently shown that, on a lap-by-lap basis, XC skiers apply a positive pacing pattern independent of both race distance and level of the skiers (Larsson and Henriksson-Larsen, 2005; Bolger et al., 2015; Formenti et al., 2015; Andersson et al., 2016; Losnegard et al., 2017). However, describing pacing patterns in terms of lap-by-lap comparisons in sports where course topography changes substantially are insufficient due to the non-constant relationship between speed, external work rate, and thereby metabolic energy demand. Therefore, describing such pacing patterns in a sport such as XC skiing, demands alternative methods where the total energy turnover could be estimated.

Previous investigations of exercise intensity and pacing patterns in XC skiing have mainly focused on sprint skiing (≤1.8 km) (Andersson et al., 2010, 2016; Sandbakk et al., 2010, 2011). The pacing pattern in XC sprint skiing has been shown to be regulated according to the terrain, with skiers applying considerably higher metabolic rates during uphill compared to flat sections of the course (Andersson et al., 2016; Sandbakk and Holmberg, 2017). This is in line with computer modeling from XC skiing and road cycling, which suggests that increased exercise intensity in uphill terrain improves performance compared to maintaining an even exercise intensity (Swain, 1997; Atkinson et al., 2007; Sundstrom et al., 2013). Moreover, estimations of the work rate during single uphill sections of competitive skiing have revealed metabolic rates of approximately 110–160% of peak aerobic power (Norman et al., 1989; Sandbakk et al., 2011), implying a considerable anaerobic energy production. However, no study has investigated anaerobic energy turnover during competitions in XC skiing. In running, Olesen (1992) has shown that the anaerobic capacity (with the maximal accumulated oxygen deficit method) during uphill running is higher compared to running on flat terrain, which may also apply to XC skiing (Andersson et al., 2016). Consequently, this may have implications for the maximal metabolic power attainable in different terrains and adds to the complexity of pacing in XC skiing. However, except for the pioneering work by Norman et al. (1989) conducted nearly 30 years ago, little information is available on exercise intensity in various terrains in distance XC skiing (>10 and 15 km for female and male skiers, respectively). Moreover, to our knowledge, no study has investigated individual energy turnover rates in different terrains during XC skiing. Since specificity is a known principle in training, a more detailed evaluation of sport specific requirements may, therefore, contribute to optimizing training and competition strategies.

One challenge when estimating energy turnover, and thereby exercise intensity, in XC skiing on snow is to control the ski friction, and thereby the external load. An alternative approach is to use roller skis, which are also used in treadmill skiing. The relationship between external workload and metabolic cost can, therefore, be determined by testing skiers during treadmill roller skiing (Sandbakk et al., 2010). Recent advances in wearable sensor technology allow tracking of athletes as a point mass model for position, speed, and acceleration, by using a differential global navigational satellite system (dGNSS), during outdoor roller skiing (Larsson and Henriksson-Larsen, 2005; Andersson et al., 2010). Using this wearable technology, the external work load can be determined (Swaren and Eriksson, 2017) and the metabolic cost of roller ski racing can be estimated to illustrate the exercise intensity during a race.

The present study, therefore, investigated pacing patterns in a 13.5 km self-paced time trial (TT), performed on roller skies on an international race course, by describing exercise intensities in flat and uphill terrain. To determine exercise intensities in the TT, external workloads were derived from accurate positioning data collected with a dGNSS system worn by the skiers. The external workloads from the TT were converted into energy demands using individual relationships between energy cost and workload collected during treadmill roller skiing. We hypothesized that: (I) During a XC distance race, skiers apply a variable pacing pattern; (II) XC skiers repeatedly perform exercise intensities exceeding their peak aerobic power during a XC distance race.

## Materials and Methods

### Subjects

Eight well-trained male XC skiers (mean ± SD: age, 23.0 ± 4.8 years; body mass, 77.1 ± 6.1 kg; height 1.84 ± 0.07 m) volunteered to participate in the study. The skiers were recruited via convenience sampling using the following criteria: (1) either active or former active competitor at a national level in Norway, (2) experienced with treadmill roller skiing and (3) familiar with the specific race course. The protocol was approved by the local ethics committee of the Norwegian School of Sport Sciences and the Norwegian Social Science Data Services (NSD). All subjects gave written informed consent in accordance with the Declaration of Helsinki. If younger than 18 years of age, parental written consent and assent from the skier were obtained.

### Experimental Overview

All skiers attended two separate sessions, separated by 11.0 ± 4.9 days. In the first session, the skiers completed a self-paced 13.5-km individual TT on an international race course to simulate a XC ski race. In the second session, individual relationships between external work rate and oxygen consumption (VO_2_), and peak oxygen consumption (VO_2peak_) and maximal accumulated O_2_-deficit in flat and uphill terrain was determined in the laboratory on a roller ski treadmill. The O_2_-demand in seven sections of the TT course was estimated by extrapolating the individual linear relationships between VO_2_ and workload using individual positioning data collected in the TT. All tests were performed on roller skis using the skate technique. The same test leaders conducted all tests.

### Time Trial

The individual TTs were carried out in the roller ski course in Holmenkollen (Oslo, Norway). The course profile resembled the actual profile of a XC-ski course used in the FIS World Cup and consisted of three identical laps of 4.5 km (height difference 51 m, maximum climb 32 m, total climb 166 m). The TTs were conducted on two separate days; six skiers completed the TT on the first day and two skiers on the second day. Before starting the TT, the skiers performed 20 min of individual warm-up, wearing the dGNSS equipment and the assigned test skis to familiarize themselves with the equipment. The skiers started the TT at 2-min intervals and were instructed to complete the TT as fast as possible. No instructions regarding pacing patterns were given. Continuous individual positioning data were recorded with a dGNSS system during the TT. Heart rate data were recorded with a separate HR monitor. In two preselected sections of the course, one uphill (S4) and one flat (S7), video recordings of the skiers were conducted to determine sub-techniques applied in these sections. In addition, the skiers verbally reported their rating of perceived exertion (RPE) on a category ratio scale (Foster et al., 2001). Air temperature during the outdoor TTs was between 8 and 16°C, and air pressure was approximately 1005 hPa. Local wind direction was northeast and southeast on the first and second day, respectively. The asphalt was completely dry on both occasions.

### Laboratory Tests

**FIGURE 1:**
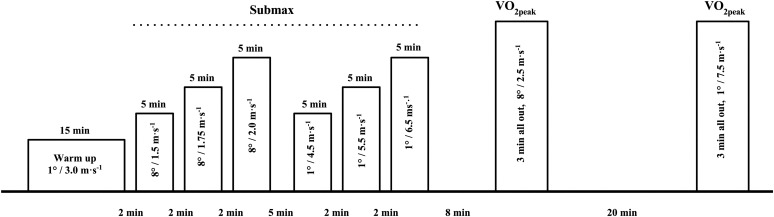
Test protocol for the treadmill submaximal workloads and performance tests. The skiers completed one subset consisting of three workloads (of 5 min each) in the V1 technique at 8° inclination and a speed of 1.5, 1.75, and 2.0 m⋅s^-1^, and one subset consisting of three (5 min) workloads in the V2 technique at 1° inclination and speeds of 4.5, 5.5, and 6.5 m⋅s^-1^. The order in which the inclined and level subsets were performed was counter-balanced between athletes. Thereafter the skiers completed two 3 min maximal performance tests at inclinations of 8° and 1° respectively. The speed was fixed the first 30 s, and thereafter the subjects controlled the speed (uphill; 0.25 m⋅s^-1^, flat; 0.5 m⋅s^-1^ increase or decrease) by adjusting their position on the treadmill. The performance tests were performed in the same order as the submaximal workloads.

The indoor tests were performed on a roller ski treadmill. Speeds, inclinations, and sub-techniques were chosen to resemble those from the outdoor TT to enable estimation of O_2_-demands during flat and uphill skiing. The indoor test protocol is illustrated in **Figure 1**. First, skiers completed a standardized 15 min warm up at 3° and 3.0 m⋅s^-1^ (∼ 60–75% of HR_peak_). The skiers then performed six submaximal workloads divided into two subsets consisting of three flat and three uphill workloads, respectively. The flat subset was meant to resemble the flat sections of the TT course, and was carried out at 1° and 4.5, 5.5, and 6.5 m⋅s^-1^ using the V2 technique (two pole plants for two ski pushes). The uphill subset was meant to resemble the uphill sections of the TT course (mean incline ≈ 8°), and was carried out at 8° and 1.5, 1.75, and 2.0 m⋅s^-1^ in the V1 technique (one pole plant for two ski pushes). The duration of each submaximal workload was 5 min, and each workload was separated by 2 min. The two subsets were separated by 5 min of passive recovery.

After 8 min of active recovery (∼60–70% of HR_peak_) the skiers completed two self-paced 3 min all-out performance tests separated by 20 min as described by Sandbakk et al. (2016). The 3 min all-out performance test has been shown to be a valid method to determine VO_2peak_ during treadmill roller skiing (Losnegard et al., 2012a). The speed was constant the first 30 s starting at 2.5 m⋅s^-1^ and 7.5 m⋅s^-1^ in the uphill (8°) and flat (1°) performance test, respectively. Thereafter the subjects controlled the speed (uphill; 0.25 m⋅s^-1^, flat; 0.5 m⋅s^-1^ increments or decrements by adjusting their position on the treadmill relative to laser beams situated in front of and behind the skier. External power, steady state VO_2_, respiratory exchange ratio (RER), ventilation (VE) and breathing rate (BR) were measured continuously during all tests. Heart rate and rating of perceived exertion (RPE) were registered, and blood lactate concentration ([La^-^]) was measured immediately after the completion of each workload. The order in which the submaximal subsets and the performance tests were performed were counter balanced. The temperature indoors was approximately 20°C, and the total duration of the indoor session was approximately 1.5 h.

### External Power

External power (*P_ext_*) on the treadmill was calculated as the sum of power against gravity (*P_g_*) and power against rolling resistance (*P_rr_*), without dGNSS equipment, previously described by Losnegard et al. (2013). External power outdoors (*P_ext_out_*) was calculated as the sum of power against gravity (*P_g_*), power against rolling resistance (*P_rr_*) and power against air drag resistance (*P_d_*):

$$P_{ext\_out}=\Sigma P=P_{g}+P_{rr}+P_{d}$$

Power against gravity was calculated as the increase in potential energy per unit time:

$$P_{g}=m\cdot g\cdot \sin\alpha \cdot v$$

where *m* represents the total mass of the skier (incl. equipment), *g* the gravitational acceleration, α the inclination of the course in degrees and *v* the skier’s speed along the track.

Power against rolling resistance (*P_rr_*) was calculated as work against rolling resistance forces per unit time:

$$P_{rr}=C_{rr}\cdot m\cdot g\cdot \cos\alpha \cdot v$$

where *C_rr_* represented the coefficient of rolling resistance of the roller skis and was measured (*C_rr_* = 0.024) before and after the project using a towing test previously described by Losnegard et al. (2011). We used the same C_rr_ for the treadmill and asphalt surface, as previous studies by our group did not find any differences using the same roller skis, asphalt track and treadmill belt (Myklebust, 2016).

Power against air drag resistance (*P_d_*) was estimated as follows:

$$P_{d}=F_{d}\cdot v$$

where *F_d_* represents the force from air drag acting on the skier. *F_d_* was estimated assuming a turbulent air flow and no environmental wind (Sundstrom et al., 2013):

$$F_{d}=0.5\cdot C_{D}A\cdot \rho \cdot v_{air}^{2}$$

where *C_D_* represents the drag coefficient, *A* the projected frontal area of the skier, *ρ* the air density, and *v_air_* the speed of the skier relative to the air. Due to the assumption of no environmental wind, *v_air_* was set equal to *v*. The drag area (*C_D_A)* was determined by scaling, as described by Sundstrom et al. (2013).

Air density (ρ) was calculated from ambient temperature measurements on site on the test day. Air pressure (*p*) was obtained from the meteorological station at Blindern (Oslo, Norway^1^). Air density *ρ* was calculated from the following equation, assuming dry air:

$$\rho =\frac{p}{R\cdot T}$$

where *R* is the specific gas constant of dry air (287.058 J⋅kg^-1^⋅K^-1^) and *T* the ambient temperature in kelvins.

### Definitions and Data Analysis

The pacing pattern was concidered variable if there were statistically significant changes in exercise intensity, expressed as O_2_ demand, throughout the TT. Conversley, the pacing pattern was concidered even if the changes in exercise intensity were statistically non-significant. VO_2peak_ in uphill and flat terrain, respectively, was defined as the highest average 30-s epoch during each of the performance tests. Peak heart rate (HR_peak_) was defined as the highest HR registered during the performance tests. Oxygen cost for each workload was defined as the average oxygen consumption between 3 and 4.5 min in each workload. ΣO_2_-deficit was calculated based on the method presented by Losnegard et al. (2012a). Gross efficiency (GE) in the submaximal workloads was defined as the ratio between external power output (W) and aerobic energy turnover rate (W) and was expressed as percentages, as described by Losnegard et al. (2014).

Two regression equations were computed for each athlete, one for flat and one for uphill skiing, assuming a linear relationship between external power and VO_2_. Individual positional data from each section were standardized according to section length (100 sample points in each section), and individual external work rate was calculated at each sample point. An estimate of the O_2_-demand was then made using the individuall regression equations and external work rates. Individual section O_2_-demand was defined as the average O_2_-demand of the 100 sample points in each section.

### Instruments and Materials

All tests were performed on Swenor Skate Long roller skis (length: 630 mm, weight incl. binding: 795 g⋅ski-1, Swenor, Sarpsborg, Norge) equipped with wheel type 2 and Rottefella Xcelerator 2.0 bindings (Rottefella, Klokkarstua, Norge). The skiers used the same pair of roller skis and their personal ski boots and ski poles (90 ± 1% of body height) in both the outdoor TT and the indoor session. Before the indoor session, the ski poles were fitted with customized treadmill ferrules. Laboratory tests were performed on a roller ski treadmill with belt dimension 3 × 4.5 m (Rodby, Södertälje, Sverige).

The dGNSS system used in the TT has previously been described and validated for kinematics (Gilgien et al., 2015) and kinetics (Gilgien et al., 2013) in alpine skiing, and has an expected accuracy < 5 cm when double difference ambiguities are fixed (Gilgien et al., 2014). The dGNSS system consisted of an antenna mounted on the skier’s helmet (G5Ant-2AT1, Antcom, United States) connected to a GPS/GLONASS dual frequency (L1/L2) receiver (Alpha-G3T, Javad, United States) placed in a small backpack. Total weight of the dGNSS system was 940 g (receiver 430 g, backpack 350 g, antenna 160 g). A stationary base station was placed in a fixed position close to the course, to facilitate differential positioning. The base station consisted of an antenna (GrAnt-G3T, Javad, United States) and a receiver (Alpha-G3T, Javad, United States). The antenna was mounted on a tripod and raised approximately 2 m above ground level. The dGNSS measurements were determined in the global coordinate system WGS84 (Universal Transverse Mercator zone 32, northern hemisphere). The dGNSS position was calculated using kinematic carrier phase double difference solutions (Gilgien et al., 2014) at 50 Hz using geodetic post-processing software (Justin, Javad, United States), and filtered using smoothing splines (smoothing parameter *p* = 0.1) weighted by their fixed/float status (Skaloud and Limpach, 2003). The position measurements were mapped onto a common trajectory based on a kinematic position tracking of the race track sampled at 1 Hz (antenna and receiver: GrAnt-G3T and Alpha-G3T, Javad, United States). The skiers’ speed v (Eq. 2) along the track was determined from the time derivative of the positions along the mapping trajectory.

During the laboratory tests, oxygen consumption was measured using an automatic ergospirometry system (Oxycon Pro, Jaeger GmbH, Hoechberg, Germany). Blood lactate concentration was measured in unhemolyzed blood from capillary fingertip samples (YSI 1500 Sport; Yellow Springs Instruments, Yellow Springs, OH, United States). The lactate analyzer and the Oxycon Pro Jaeger Instrument were calibrated according to the instruction manual as described in detail previously (Losnegard et al., 2011). Body mass and mass including equipment were measured before the TT and the treadmill test (Seca model 708; Seca, Hamburg, Germany).

Rating of perceived exhaustion was evaluated using a category ratio RPE scale (0–10) validated by Foster et al. (2001). Heart rate was recorded using the athletes’ personal training computers. Video was recorded with two Canon HF100 video cameras (frame rate = 25 Hz, Canon Inc., Tokyo, Japan). Environmental temperature, air pressure, and wind data were retrieved from local weather stations (met.eklima.no, Meteorological Institute of Norway, Oslo, Norway).

### Statistics

Data are presented as the mean ± standard deviation (SD) unless otherwise stated. Normality of the data was assessed using the Shapiro–Wilks test of normality (α = 0.05). Outliers were assessed by inspection of boxplots and by examination of studentized residuals for values greater than ± 3. Paired sample *T*-tests were used to detect statistical differences in average speed, VO_2peak_, ΣO_2_-deficit, HR_peak_, [La^-^], VE and RPE between the 3-min all-out performance tests, and between GE during flat and uphill submaximal skiing. One-way repeated measures ANOVAs, with Bonferroni correction for multiple comparisons, were conducted to determine whether there were statistical differences in average lap speed, section O_2_-demand, section external power and section speed between laps, and in average section O_2_-demands between sections in the TT. Average HR between laps and RPE during the TT failed the assumption of normality and were analyzed with related samples Friedman’s tests, with Bonferroni correction for multiple comparisons. Pearson’s Product Moment Correlation Analysis was applied for correlation analysis between VO_2_ and external work rate on the treadmill. The strengths of correlation (*r*) were interpreted as follows: correlation coefficient (*r*) < 0.1 trivial; 0.1–0.3, small; 0.3–0.5, moderate; 0.5–0.7, strong; 0.7–0.9, very strong; and 0.9–1.0, almost perfect (Hopkins, 2002). An α level of *p* ≤ 0.05 was considered significant, and *p* ≤ 0.10 was considered a tendency. All calculations were performed in MATLAB R2016a (MathWorks, Inc., Natick, MA, United States), and statistical analyses were performed in SPSS Statistics (IBM Corp., Armonk, NY, United States).

## Results

### Laboratory Tests

**FIGURE 2:**
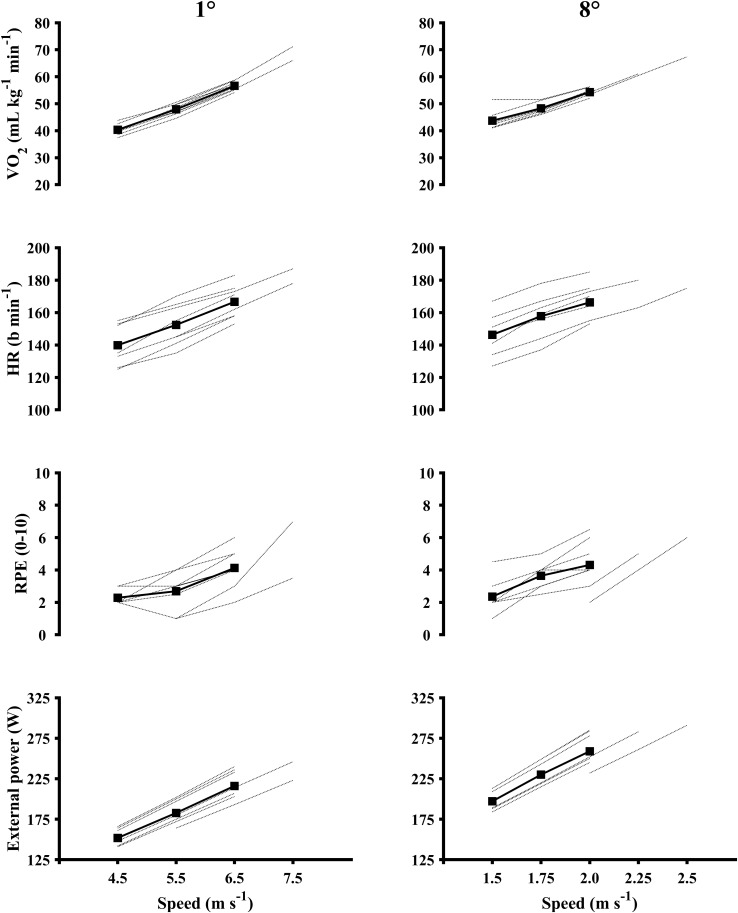
VO_2_, HR, RPE and external power during the submaximal level and inclined workloads on the roller ski treadmill. Square and solid line: average data. Dotted lines: individual data. (*n* = 8). Subject 2 completed one extra workload at 7.5 m⋅s^-1^ (total number of level workloads: 4) and one extra workload at 2.25 m⋅s^-1^ (total number of inclined workloads: 4). Subject 8 started the level submaximal workloads at 5.5 m⋅s^-1^ and the inclined submaximal workloads at 2.0 m⋅s^-1^.

Oxygen consumption during the flat and uphill submaximal workloads corresponded to 57 ± 5%, 66 ± 6%, 78 ± 6%, and 62 ± 9%, 69 ± 8%, 76 ± 8.0% of VO_2peak_, respectively (**Figure 2**). Heart rate was 76 ± 7%, 83 ± 7%, 91 ± 5%, and 80 ± 7%, 86 ± 7%, and 90 ± 6% of HR_peak_, respectively. Correlations between VO_2_ and external power were large to very large during flat [*r*(25) = 0.86, *p* = 0.001] and uphill [r(25) = 0.90, *p* = 0.001] roller skiing, respectively. There were no differences in GE between workloads at the same inclination, but GE was significantly different between flat [(14.4% ± 0.6%) = 0.9%] and uphill (17.8% ± 0.7%) with a mean differense of 3.4% [*t*(25) = -26.480, *p* < 0.001].

Physiological variables from the flat and uphill performance tests are presented in **Table 1**. There were no significant differences in VO_2peak_, HR_peak_, [La^-^], RER or RPE between the two conditions. However, the ΣO_2_-deficit was 34.8% higher in the uphill compared to the flat performance test [t(7) = -5.676, *p* = 0.001].

**Table 1:** Average speed and physiological responses during the flat (1°) and uphill (8°) 3 min all-out performance tests on the roller ski treadmill.

	1°		8°		
			
Variable	Mean ± *SD*	Range	Mean ± *SD*	Range	*p*
Average speed (m⋅s^-1^)	8.04 ± 0.36	7.58 - 8.62	2.92 ± 0.28	2.57 - 3.37	< 0.001
VO_2peak_ (mL⋅kg^-1^⋅min^-1^)	72.7 ± 5.3	64.9 - 81.5	72.3 ± 6.2	64.2 - 82.7	0.411
ΣO_2_-deficit (L)	4.6 ± 0.5	3.8 - 5.3	6.2 ± 0.6	5.3 - 7.0	0.001
HR_peak_ (b⋅min^-1^)^a^	182 ± 5	174 - 188	183 ± 6	173 - 190	0.365
[La^-^] (mmol⋅L)^b^	7.6 ± 1.0	6.1 - 8.9	6.9 ± 1.6	4.9 - 9.6	0.138
VE (L⋅min^-1^)	200.5 ± 10.8	175.1 - 210.7	188.6 ± 14.1	169.0 - 211.3	0.037
RPE (0–10)	9 ± 1	8 - 10	9 ± 1	7 - 10	0.504


**n* = 8, ^*a*^*n* = 6, ^*b*^*n* = 7.*

### Time Trial Characteristics

**FIGURE 3:**
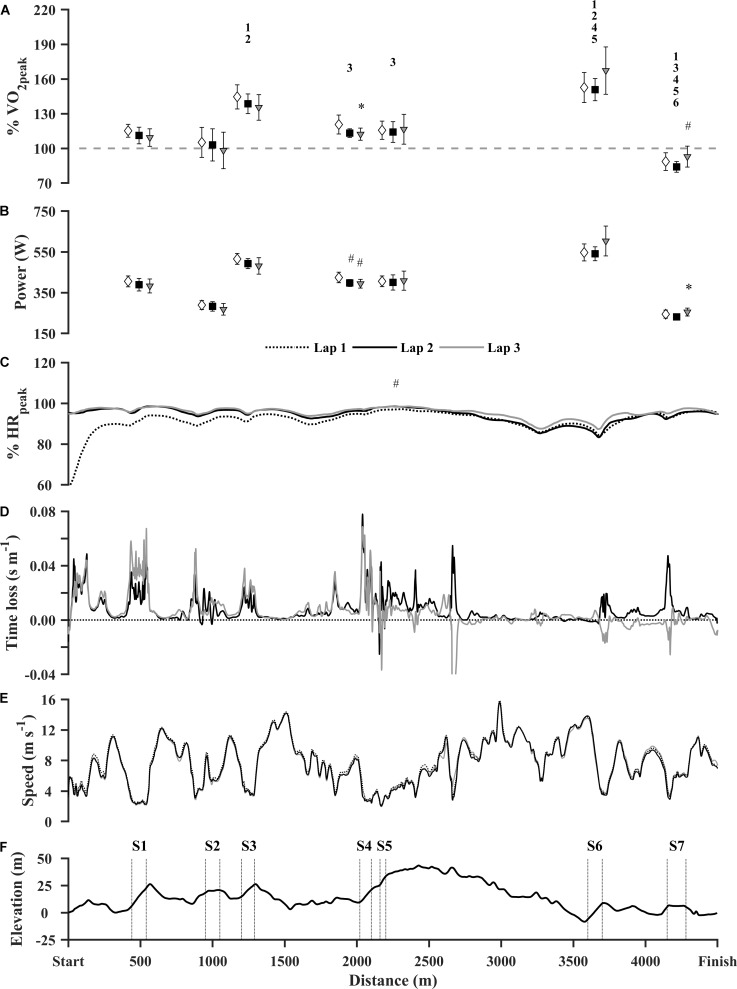
Physiological and performance characteristics from the 13.5 km time trial. **(A)** Section O_2_-demands (%VO_2peak_), **(B)** external power (W), **(C)** continuous HR (b⋅min^-1^), **(D)** time loss compared to lap 1 (s⋅m^-1^), **(E)** speed (m⋅s^-1^) and **(F)** course profile. Due to challenging conditions at ∼2700 m the GNSS-solution were poor. Hence, the spikes in time loss does not represent the real difference. Exercise intensity and external power are presented as mean ± 95% confidence intervals. Open diamond: lap 1. Closed square: Lap 2. Gray triangle: lap 3. Numbers indicate a significant difference (*p* < 0.05) in average section O_2_-demand across the 3 laps from the section with the corresponding number. #Significantly different from lap 1. ^∗^Significantly different from lap 2. (*n* = 7).

Mean TT finishing time was 33:25 ± 01:38 mm:ss, corresponding to an average speed of 6.7 ± 0.3 m⋅s^-1^. Average lap speed changed significantly during the TT [*F*(2,12) = 7.371, *p* = 0.008], with a significant reduction in speed between lap 1 and lap 2 (-0.2 m⋅s^-1^, *p* = 0.044). There were no significant differences between lap 1 and lap 3 or between lap 2 and lap 3 (**Figure 3E**). Comparing speeds within each setion revealed significant changes in S4 [*F*(2,12) = 14.765, *p* = 0.001], with a a reduction in speed from lap 1 to lap 2 and from lap 1 to lap 3, with a mean difference of 6.1% (*p* = 0.048) and 7.1% (*p* = 0.003), respectively. In addition, there were significant changes in S7 [*F*(2,12) = 5.915, *p* = 0.016], with an increase in speed from lap 2 to lap 3, with a mean difference of 5.0% (*p* = 0.035). Continuous time loss and speed are presented in **Figures 3D,E**.

External power varied between ∼230 and 600 W (**Figure 3B**). Section O_2_-demands from each lap are presented in **Figure 3A**. Section O_2_-demand varied significantly in S4 [*F*(2,12) = 14.163, *p* = 0.001] and S7 [*F*(2,12) = 6.802, *p* = 0.011]. In S4, there was a tendency to a reduction in O_2_-demand between lap 1 and lap 2 (7.4%, *p* < 0.051), and a significant reduction in O_2_-demand between lap 1 and lap 3 (8.3%, *p* = 0.005). In S7, there was an increase in O_2_-demand (8.9%, *p* = 0.034) between lap 2 and lap 3. There were no significant differences in O_2_-demands between laps in S1, S2, S3, S5, or S6. Comparing average O_2_-demands (over three laps) between sections, showed that section significantly influenced O_2_-demands [*F*(6,36) = 65.816, *p* < 0.001, *post hoc* results presented in **Figure 3A**], which ranged from 89 to 157% of VO_2peak_.

The average HR during the TT was 94 ± 3% of HR_peak_ (**Figure 3C**). There were significant changes in average HR between laps [χ^2^(2) = 8.3, *p* = 0.016]. From lap 1 (Mdn = 92% HR_peak_, IQR = 4%) to lap 3 (Mdn = 95% HR_peak_, IQR = 2%) (*p* = 0.016). There were, however, no statistical differences in average HR between the other laps.

Rating of perceived exertion changed significantly between the different laps of the TT [χ^2^(2) = 26.393, *p* < 0.001], with differences between S4 lap 1 (Mdn = 6.0, IQR = 3.0) and S4 lap 3 (Mdn = 8.0, IQR = 1.0) (*p* = 0.009), between S4 lap 1 (Mdn = 6.0, IQR = 3.0) and S7 lap 3 (Mdn = 9.0, IQR = 1.5) (*p* = 0.002) and between S7 lap 1 (Mdn = 7.0, IQR = 2.0) and S7 lap 3 (Mdn = 9.0, IQR = 1.5) (*p* = 0.015). The preferred sub-techniques in S4 and S7 were V1 and V2, respectively.

## Discussion

The present study investigated pacing patterns by describing exercise intensities in flat and uphill terrain during a self-paced roller ski time trial. The principal findings were that in a XC distance race: (I) the skiers frequently applied exercise intensities exceeding their peak aerobic power and exercise intensity was higher in uphill compared to flat terrain; (II) the skiers applied a variable pacing pattern, evidenced by significant changes in exercise intensity; (III) while peak aerobic power in flat and uphill skiing were similar, the ΣO_2_-deficit during uphill skiing were greater compared to flat.

### Pacing Pattern and Exercise Intensity Distribution

To our knowledge, this is the first investigation to use positional data, combined with physiological measurements, to determine the individual oxygen demand in multiple sections of a self-paced XC ski race. As evident by the considerable variations in O_2_-demand (**Figure 3A**), the skiers applied a variable pacing pattern throughout the TT, which is in accordance with earlier observations from competitive XC sprint skiing (Andersson et al., 2010, 2016). It has been suggested that athletes apply a variable pacing pattern in an attempt to maintain the same exercise intensity throughout a race (Abbiss and Laursen, 2008). In this study, however, even though the speed was substantially lower in the uphill sections of the course, O_2_-demands were considerably higher compared to the flat sections (**Figure 3A**). This implies that the skiers did not maintain an even exercise intensity, but rather repeatedly increased the intensity in the uphill sections. Moreover, the large variations in exercise intensity and the disassociation between exercise intensity and speed between different terrains emphasizes that describing pacing patterns exclusively by inter-lap variations in speed is insufficient (Abbiss and Laursen, 2008). At least, this is true for endurance sports events where substantial variations in the topography exist.

Comparing the exercise intensity in S1 and S6, two sections of approximately the same length and inclination, the O_2_-demand was approximately 50% higher in S6 (**Figure 3A**). This difference in exercise intensity can be explained by the fact that prior to S6, the skiers performed approximately 1 km of downhill terrain (**Figure 3F**). This allowed the skiers to arrive at S6 in a partially recovered state abel to apply a greater amount of anaerobic work in this section. Further, S6 was followed by relatively even terrain, thus implying, that the skiers had a less strenuous part of the course ahead of them after S6. It was also evident that when the length and inclination of the uphill section increased (S4 and S5), the skiers reduced the intensity (∼115% of VO_2peak_) compared to the shorter and less steep sections (S3 and S6, 140–160% of VO_2peak_). Furthermore, as the skiers approached the end of the race, they increased the exercise intensity (S6 and S7). This increase in exercise intensity has been described as the “endspurt phenomenon,” and has been explained by a reduction in uncertainty regarding the remaining work (Tucker, 2009). Taken together, these observations imply that skiers modify their exercise intensity and hence their pacing according to the terrain and their current position in the course.

### Exercise Intensity in Uphill Terrain

The rationale for applying higher exercise intensities in uphill than in flat terrain, and thus a variable pacing pattern, involves at least three factors. First, the quadratic increase in air drag with speed implies that a substantial fraction of the increase in propulsive power is dissipated to overcome the increase in air drag resistance. In the flat sections, air drag resistance accounted for approximately 50% of the external work. In contrast, in the uphill sections where the speeds were low, the work against air drag was negligible (∼3% of P_ext_out_). Since performance in uphill terrain is a determinant of overall performance in XC skiing (Andersson et al., 2010; Sandbakk et al., 2011), the most rational choice is to increase work rate in the uphill parts of the course.

Second, repeated periods of supramaximal intensities are possible because of the downhill sections, where the skiers are propelled mostly by gravity. Since the anaerobic capacity is limited, either cessation of work or a reduction in work rate to a level that can be met by aerobic metabolism must occur following a period of supramaximal work rates (Gastin, 2001). In the present study, we did not estimate the O_2_-demand of downhill skiing, but previous estimations suggest that it is approximately 40–60% of VO_2max_ (Sandbakk and Holmberg, 2017). Further, VO_2_ values of approximately 65% of VO_2max_ during downhill skiing have been reported during competition (Welde et al., 2003). Therefore, it seems reasonable to assume that there is a sufficient drop in O_2_-demand during downhill skiing to recover at least some of the O_2_-deficit attained. This is exemplified by the differences in O_2_-demands between S1 and S6, as described above.

Third, the possibility of repeatedly attaining an O_2_-deficit and at least partially recovering from it without a decrease in speed separates XC skiing from other endurance sports, such as running, track cycling and speed skating. In these endurance sports athletes can maintain an intensity relying on a high contribution of anaerobic metabolism for only a limited time, without reducing the speed (Gastin, 2001). To our knowledge, the present study is the first to directly compare the VO_2peak_ and the ΣO_2_-deficit of XC skiers during both flat (1°) and uphill (8°) skiing. A novel finding was that the ΣO_2_-deficit was significantly greater in the uphill performance test compared to the flat. Such a difference has previously been reported in treadmill running and has been attributed to a greater amount muscle mass being active when running uphill (Olesen, 1992). The difference in O_2_-deficit between flat and uphill terrain has implications for the duration and magnitude of supramaximal work rates in different type of terrains. Since the peak aerobic power was similar in flat and uphill terrain, the total metabolic power attainable in different terrains is determined by the anaerobic energy turnover. In addition, the ΣO_2_-deficit seems to be an important factor for training-induced seasonal changes and thereby performance in elite distance XC skiers (Losnegard et al., 2013). Taken together, even though the duration of a XC distance race is relatively long (>30 min), and the relative contribution from anaerobic metabolism is low, the ability to repeatedly apply work rates covered by a high anaerobic turnover seems to be a crucial factor for performance in elite XC skiing, which to date is not fully understood.

### Heart Rate and Exercise Intensity

From a practical standpoint, HR is a widely used tool to describe exercise intensity in endurance sports (Achten and Jeukendrup, 2003). In the present study, HR remained high for most of the race (>90% of HR_peak_) (**Figure 3C**), which is in accordance with previous observations in XC skiing (Mognoni et al., 2001; Formenti et al., 2015). Our results also show that HR to some extent reflects the exercise intensity in various parts of the course. However, the ability of the HR to reflect rapid-intensity transients and supramaximal exercise intensities is limited due to the temporal dissociation between HR, VO_2_ and work rate during high-intensity exercise (Buchheit and Laursen, 2013; Bolger et al., 2015). This is supported by our observations when comparing HR and O_2_-demands in the different sections of the course (**Figures 3A,C**). While a considerable variation in O_2_-demand was evident, there were relatively small variations in HR. Hence, HR is not suitable for describing exercise intensity in XC skiing competitions.

### Methodological Considerations

In the present study, we assumed that the linear relationship between external power and O_2_ cost established during the submaximal workloads (∼55–80% of VO_2peak_) also applied to maximal and supramaximal workloads observed in the TT (∼85–160% of VO_2peak_). This relationship is well established at workloads below the lactate threshold (Bassett and Howley, 2000; Noordhof et al., 2010). However, it is debated whether this linearity also applies to workloads above the lactate threshold (Noordhof et al., 2010). Furthermore, taking both the duration of the TT and the intensities applied into account, a VO_2_ slow component must be expected (Jones et al., 2011). This could result in a reduction in GE and an increase in the energy cost of maintaining the same external workload. We did not quantify alterations in the GE during the TT. Thus, we may potentially have underestimated the actual O_2_-demand, at least in the later parts of the TT. These considerations should be taken into account when interpreting the results.

The air drag resistance acting upon a XC skier is a complex mechanism, affected by changes in body position, sideways movement and clothing (Spring et al., 1988; Leirdal et al., 2006). In the present study, we assumed that the upright position, described by Spring et al. (1988) represented the average body position of the skier. Because of the quadratic behavior of the air drag resistance, the body position of the skier potentially has a significant influence on the external power at high speeds. However, most of the sections in the current study were uphill (n_uphill_ = 5). Hence, the speeds and the relative contributions of air drag resistance were small (∼3%) and should not have influenced the results. Moreover, environmental wind conditions may influence the relative air flow and affect the air drag resistance. In the present study, no environmental wind was assumed in the calculations as wind conditions on both test occasions were negliabe.

Power against rolling resistance on the treadmill was quantified using the method described by Losnegard et al. (2012a). The limitations of this approach were discussed by the authors and can be attributed to changes in the normal force and the orientation angle of the roller ski in relation to the direction of travel. However, the effects were small, and would only have minor implications for the estimation of total external power.

The different ski-skating techniques applied in the flat and the uphill sections could potentially influence the VO_2peak_ and O_2_-deficit. However, Losnegard et al. (2012b) reported no differences in performance, VO_2max_ or O_2_-deficit between V1 and V2 at steep inclines (6–8°) and differences in applied sub-techniques were considered to have minimal impact on the results.

A strength of the present study is the use of individually estimated O_2_-demands, which to date has not been applied in simulated races outdoors. Thus, the presented method makes it possible to describe not only the external workload but also the physiological workload imposed on skiers in a XC ski race. However, it should be noted that the results are based on estimations and not direct measurement of the O_2_-demand. To our knowledge, direct methods to measure anaerobe turnover during field tests are at present not well developed. Moreover, the positional data were collected during a self-paced time trial conducted on a world-cup race course, implying that the results are of high relevance to determine performance in international competitions.

### Future Studies

In this study, measurements of GE were restricted to steep inclines (8°) or flat (1°) terrain, which limits inferences about inclinations between these two conditions. Hence, further knowledge of how metabolic rate depends upon inclination and/or skiing speed would be useful for assessing exercise intensity throughout a ski race. Moreover, we did not measure aerobic energy consumption during the TT. Such measurements would enable calculation of the O_2_-deficits attained throughout the race, thereby providing a more detailed view of the rates of aerobic and anaerobic energy production.

### Practical Application

Even though a high aerobic energy turnover always has been mandatory for performance in elite XC skiing, our results also suggest that the ability to repeatedly utilize a high anaerobic energy turnover is of great importance in distance XC skiing. Furthermore, we revealed that the O_2_-deficit is terrain specific, implying the importance to develop this capacity specifically for different terrains. Further, the observation that HR did not accurately reflect exercise intensity during the TT suggests that athletes and coaches should consider other methods to quantify exercise intensities during competitions and high intensity training sessions with rapid changes in terrain. Finally, the wearable GNSS units used in the current study provides researchers a valuable tool for detailed analysis of performance in XC skiing. As the technology improves and smaller units become available, these units could also become a valuable tool for coaches and athletes when evaluating training and competition strategies.

## Conclusion

The present study investigated energy demands flat and uphill terrain during a self-paced roller ski time trial. This was accomplished by applying a novel approach combining accurate positioning data collected with a wearable dGNSS system, with individual physiological data collected during treadmill roller skiing. Our findings revealed that XC skiers repeatedly applied exercise intensities exceeding their maximal aerobic power during a XC distance race. Hence they applied a variable pacing pattern. Furthermore, the ΣO_2_-deficit was considerably higher during uphill skiing compared to flat which has implications for the duration and magnitude of supramaximal work rates that can be applied in different types of terrain.

## Author Contributions

ØK, MG, and ØG analyzed the data. All authors contributed to the design of the study, colletion of data, and writing.

## Conflict of Interest Statement

The authors declare that the research was conducted in the absence of any commercial or financial relationships that could be construed as a potential conflict of interest.

## Footnotes

1 eklima.net
